# Ethnopharmacologic survey of medicinal plants used to treat human diseases by traditional medical practitioners in Dega Damot district, Amhara, Northwestern Ethiopia

**DOI:** 10.1186/s13104-017-2482-3

**Published:** 2017-04-18

**Authors:** Muluken Wubetu, Tefera Abula, Getye Dejenu

**Affiliations:** 1 Pharmacy Department, Debre Markos University, Gojjam, Ethiopia; 2 School of Pharmacy, Addis Ababa University, Addis Ababa, Ethiopia; 3 Public Health Department, Debre Markos University, Gojjam, Ethiopia

**Keywords:** Medicinal plants, Traditional medical practitioner, Ethnopharmaclogy

## Abstract

**Background:**

One of the services that plants provide for human beings is their wider medicinal application. Although it is not fully assessed, the practice and wider use of traditional medicine is frequent in Ethiopia. Studies conducted previously are confined to the perceptions of modern and traditional health practitioners about traditional medicine. A total of 45 informants were selected purposefully from the study area. For collecting the data, semi-structured interviewees, observation and field walks were employed from August 10 to September 30/2014. To summarize the information, descriptive statistical methods were applied.

**Results:**

Sixty species of medicinal plants distributed in 42 families were collected and identified applied locally for the treatment of 55 human disorders. The most commonly treated ones were evil eye, malaria, wound, peptic ulcer disease and rabies. According to this study, leaves were the commonly used plant parts (36.5%) and 39% of the preparations were decoctions. Oral route, 43 (44%) was the commonly used route of application whereas most (54.8%) remedies were administered only once. Fourteen percent of preparations caused vomiting in addition most (40.4%) of the formulations was contraindicated for pregnant patients. Only seventeen percent of the formulations possessed drug food interactions. Most preparations were stored within clothes, 31 (29.8%). There exists a high (ICF = 0.8) evenness of plant use among healers for treating respiratory problems. *Alliumsativum* (FI = 0.75) for evil eye, *Phytolacca dodecandra* (FI = 0.8) for rabies and *Croton macrostachyus* (FI = 0.78) for treating malaria were medicinal plants with highest fidelity levels showing consistency of knowledge on species best treating power. This study also documented that drought, overgrazing and firewood collection are major threats.

**Conclusion:**

Dega Damot district is loaded in its medicinal plant diversity and indigenous knowledge though plants are highly affected by drought, overgrazing and firewood collection. Therefore awareness activities must be created among the district’s population by concerned governmental and nongovernmental organizations about the value of medicinal plants and conservation of these plants. The healing potential and associated adverse issues of the claimed medicinal plants should be assessed before proposing for a broader utilization.

**Electronic supplementary material:**

The online version of this article (doi:10.1186/s13104-017-2482-3) contains supplementary material, which is available to authorized users.

## Background

According to world health organization, traditional medicine (TM) is the sum total of the knowledge, skills and practices based on the theories, beliefs and experiences indigenous to different cultures and nations. It is used in the maintenance of health, prevention, diagnosis, or treatment of disorders [1, 2]. Under TM, health practices, remedies, approaches, and beliefs incorporating plant, animal and mineral products, spiritual therapies are all included [3]. Traditional medicine is popular in the developing world and its use is rapidly spreading in the developed nations. In China, traditional herbal preparations account for 30–50% of the total drug consumption. In Ghana, Mali, Nigeria and Zambia, the first choice for 60% of children with high fever resulting from malaria is the use of herbal medicines. In Ethiopia up to 80% of the population uses TM due to the cultural acceptability of healers and local pharmacopeias, the relatively low cost of TM and difficult access to modern health facilities [4].

In Ethiopia, TM plays both preventive and curative roles. Vegetables are the abundant sources of traditional remedies. Various parts of plants like leaves, flowers, seeds, bark, sap and roots are used. Honey, butter, and sheep fat are TM sources from animals. In Ethiopia, traditional medical practitioners put much emphasis on the supernatural force as a source of wisdom for healing various illnesses. Even though practitioners practically deal with tangible problems like bone setting, simple traditional surgery, historical evidence shows that there were many prayers for the prevention and cure of various ailments [1, 5–8]. Despite its continued use over many countries, its popularity and extensive use, TM has not been officially recognized in most countries. As a result, training and research in this area have not been conducted intensively on the various aspects of TM. The safety and efficacy of data on TM are not sufficient to meet the criteria required to support its worldwide use [4, 5, 9, 10].

In Dega Damot district about 90% of the population relies on traditional health products (unpublished data from the district) for primary health care aspects. There have been no studies conducted in the study area on the use and practice of TM. For policies regarding TM ultimately geared towards integration of TM into the national health service to be formed and implemented, results of this study will be able to protect the interest of those making use of this health care option.

## Methods

### Description of the study district

**Fig. 1:**
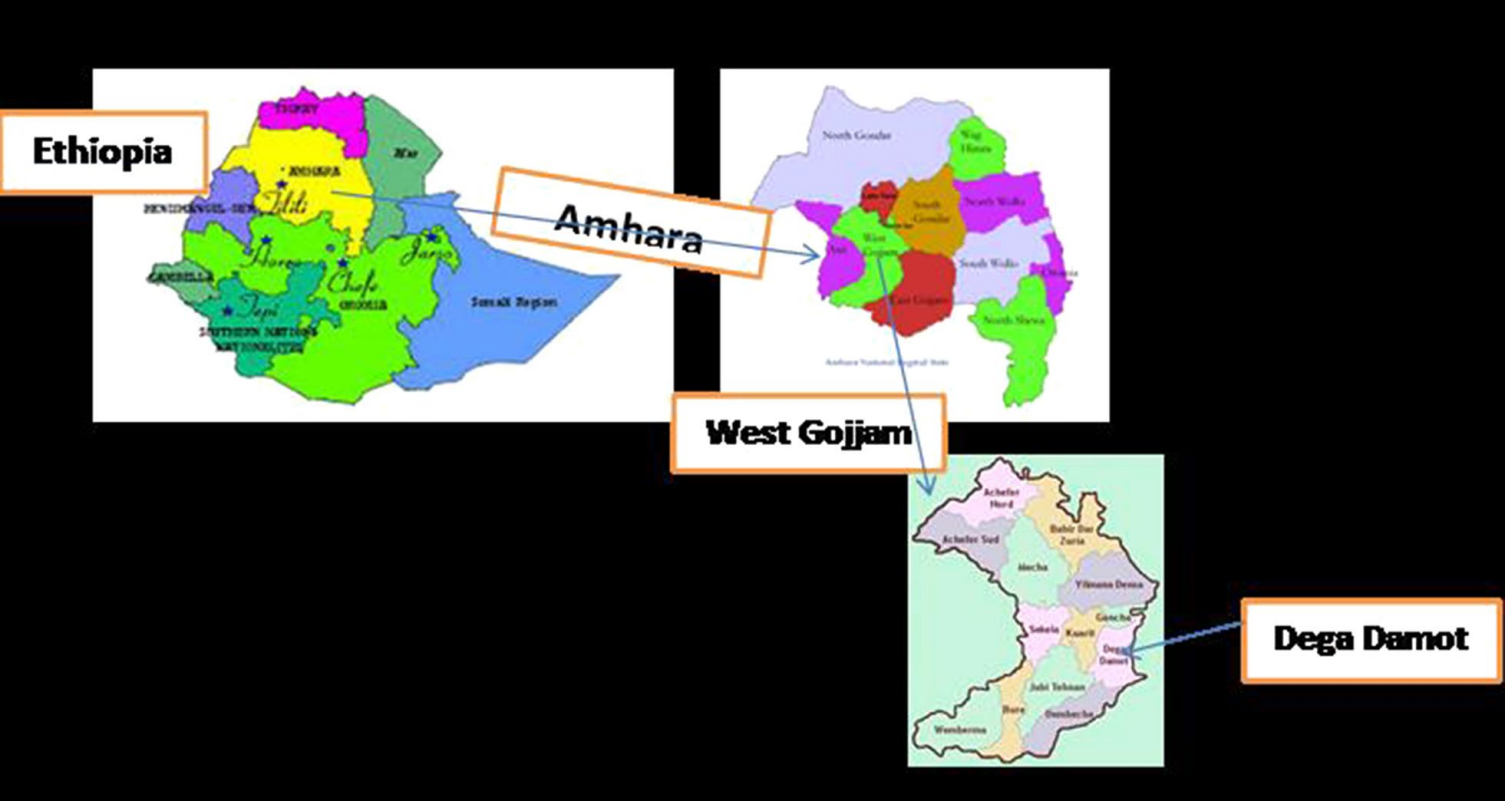
Map of the Dega Damot district (the officials who gave permission to use the respective maps were: personnel of Dega Damot district communication office, west Gojjam zonal communication office and Amhara regional state communication beau rue)

This study involved traditional medical practitioners residing in Dega Damot district, Northwestern Ethiopia. It shares borders with the districts of Bibugn in the north, Dembecha in the east, Kuarit and Hulet eju enesie in the west and Jabitehnan in the South (Fig. 1). The district’s administrative town, Feres Bet, is located at about 400 km north western of the capital, Addis Ababa. According to 2013 data, the population of the district is about 170, 575. The district is administratively divided into 32 kebeles and Amharic which belongs to the Semitic language family is the language of the population. In Ethiopia, Kebele is the smallest administrative unit. In the district, barley, maize, potato and wheat are the main crops cultivated, off which, potato is exported to neighboring towns of the Amhara region like Burie, Bahirdar and Gondar.

According to 2004–2013 rainfall data, the District has a high rainfall distribution between July and August and a smaller rainfall between January and May. The mean monthly rainfall and mean annual rainfall of the District are 60.24 and 708.54 mm, respectively (National Metrological Service Agency, Bahirdar Branch Office, unpublished data). According to data from the health personnel of the district, the top ten diseases in 2014 are malaria, diarrhea, helmenthiasis, pneumonia, acute upper respiratory diseases, dyspepsia, typhoid, eye infection, urinary tract infection and skin infection respectively.

### Selection of study subjects

Data were collected from the traditional medical practitioners (TMPs) who were purposively chosen with the help of community leaders and local authorities. The informants selected were the most knowledgeable ones as recommended by community leaders and local authorities who involved in the selection process. The ages of the TMPs ranged between 22 and 80 years. A total of 45 (40 male and 5 female) TMPs were included in this study from August 10 to September 30/2014.

### Data collection techniques

Semi-structured interviews, observation and field walks were used to collect the research data. To collect information about local names of plants used, their threats, part(s) used, preparations methods, routes of remedy administration and diseases treated, individual interviews were conducted (Additional file 1). Interviews were carried out in Amharic, language that is spoken by the practitioners. For claimed plant, specimen was collected, and identified and voucher was reserved at University of Gondar. Field observations were performed to document habitat of each medicinal plant. As this study has been conducted on wild plants, permission was mandatory to perform the survey. Hence, Dega Damot district agricultural office was informed and asked permission to conduct the study and collect the medicinal plants. The study was also ethically approved by the Graduate Program Evaluation Committee of the College of medicine and health sciences, University of Gondar. Prior to the initiation of the data collection, the objective of the survey was clarified to the TMPs, verbal consent was obtained from them. Letter of collaboration was sent to district officials of the study area and biology department at University of Gondar.

### Data analysis

Descriptive statistic procedures like percentage and frequency distribution were applied for analyzing and summarizing the data. To check the level of homogeneity among information provided by traditional practitioners, the informants’ consensus factor, ICF [11] was computed

$$[{\text{ICF}} = {\text{Nur}} - {\text{Nt}}/({\text{Nur}} - 1)]$$

where, Nur = number of use reports from informants for a particular plant-use category; Nt = number of taxa or species that are used for that plant use category for all informants. ICF Values vary between 0 and 1, where ‘1’ represents the highest level of consensus. The fidelity level (FL), which shows for the percentage of informants claiming the use of a certain plant species for the same major purpose, was calculated for the commonly reported disorders as:

$${\text{FL}}\;(\% ) = ({\text{Np}}/{\text{N}}) \times 100$$

where: Np = number of traditional practitioners that claim a use of a plant to treat a certain disease; N = number of informants that use the plants as a remedy to treat an ailment [12].

## Results

### Socio-demographic data of the informants

Totally, 45 TMPs out of which 40 (88.8%) and 5 (11.2%) males and females, respectively, were involved in this study and 55.6% were illiterate. Most of them were married (86.7%) and 37.8% were older than 56 years. Thirty-one (68.9%) were farmers and all TMPs were Ethiopian orthodox tewahido Christian followers.

Forty percent of healers indicated that they acquired their healing wisdom from their family, whereas 26.6% assumed it as a gift from God. Other sources of wisdom are religious Institutions (22.2%) and preceding sickness and corresponding use (11.2%). About 67% of the practitioners had practiced their healing activities for more than 25 years.

### Diseases treated and medicinal Plants used

**Fig. 2:**
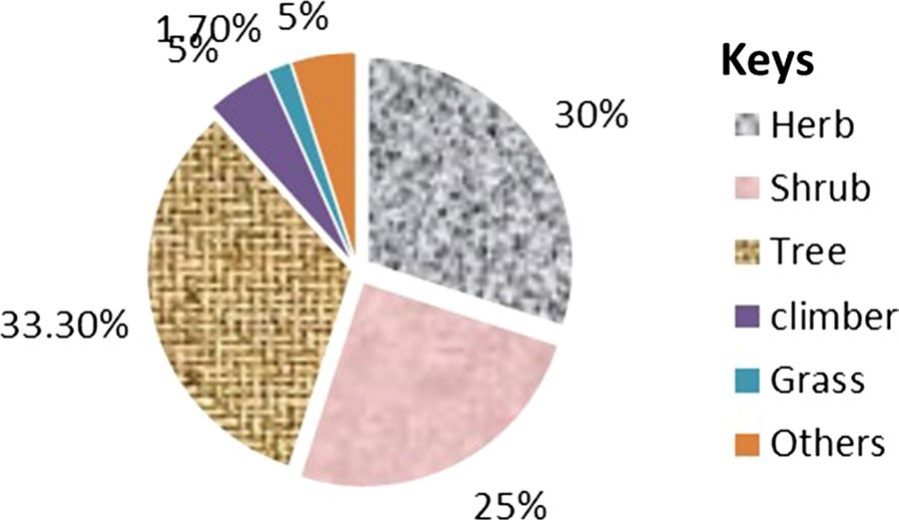
Frequency distribution of growth form of medicinal plants

About 55 human diseases are treated by TMPs of the district. The most commonly treated ones being evil eye, malaria, wound; peptic ulcer disease and rabies (Table 1).This study revealed that about 60 plant species find applications by the TMPs of the district. Those plants were identified and distributed in 42 families. Families, Gramineae and Solanaceae each accounts 4 (9.5%) medicinal plants followed by Fabaceae and Leguminosae, 3 (7%) each. Most of the plants collected and identified from the study area were trees (40%), followed by herbs (30%) and shrubs (25%) and (Fig. 2).

**Table 1:** Medicinal plants used to treat human diseases

Scientific name	Family name	Voucher no.	Amharic name	Habit	Part used	Used for	Preparation, dose and application
*Acacia Senegal (*L.*) Wild.*	Leguminosae	MW-053	Grar	Tree	Resin	Stabbing pain	Powder of resin taken mixed with molten butter
*Agrostis semiverticillata* (Forssk.) *Christm.*	Gramineae	MW-049	Serdo	Grass	Leaf	Tinea decalvans	Fresh pulverized leaf is applied once daily
*Albizia gummifera* (*J.F.Gmel) C.A.Sm*	Leguminosae	MW-039	Sesa	Tree	Bark	Rectal prolapsed	About 80 ml of Powder of bark mixed with little water (decoction) taken once daily
*Allium sativum* L.	Alliaceae	MW-001	Nech Shinkurt	Bulb	Seed	Evil eye	Crushing the seed with seeds of *Lepidium sativum* L. and *Ruta chalepensis* L. and inhale it
					Fruit	Common cold	Inhale the smell of the fruit
					Fruit	Malaria	Crushing the fruit and boil it, finally drink it with much amount of milk for 1 day
					Seed	Dry cough	Crushing the seed and drink with adulterated butter
*Aloe* *pulcherrima Gilbert* *& Sebsebe*	Aloaceae	MW-002	Eret	Tree	Latex	Wound	Applying the latex to the wound for 2 days
*Artemisia* *afra* *Jack.* *ex* *Wild.*	Asteraceae	MW-003	Chikugn	Shrub	Leaf	Common cold	Inhaling the smell of the leaf
					Leaf	Urine retention	Powder of leaf taken once mixed with mead
					Leaf	Haematuria	Milk decoction of leaf taken once
*Avena sativa* L.	Poaceae	MW-004	Ankerdad	Grass	Seed	Wound	Drying the seed then crushing, then apply on the wound till the wound cures
*Bersama abyssinica* Fresen.	Melianthaceae	MW-005	Azamira	Tree	Leaf	Ascariasis	Crushing the leaf and drink it
*Brucea antidysenterica J. F. Mill.*	Simaroubaceae	MW-006	Abalo	Tree	Root	Evil eye	Crushing its root with the roots of *Pterolobium* *stellatum* (*Forssk.*) *Brenan, Carissa spinarum* L. and *Clausena anisata (Wild) Benth*. and inhale it
					Seed	Cutaneous leshmaniasis	Crushing the seed and apply on the infected area
					Leaf	Leprosy	Handful of fresh leaf grounded to make a paste and to it add small quantity of honey or butter and it is applied externally until cure
*Capsicum annuum* L.	Solanaceae	MW-007	Berbere	Shrub	Leaf	Anthrax	Crushing the leaf with leaves of *Vernonia amygdalina Del*. and eat it on empty stomach once
					Leaf	Infertility	Small quantities of fruit chewed and swallowed once
*Carissa spinarum* L.	Apocynaceae	MW-008	Agam	Shrub	Root	Evil eye	Crushing the root with the fruit of garlic and the fruit of *Ruta chalepensis* L., finally inhale it
					Root	Snake bite	Crushing the root and bandage on the site of bite for 1 day
*Citrus aurantifolia*.	Rutaceae	MW-036	Lomi	Tree	Leaf	Hypertension	Crushing the leaf and drying it, finally drinking it as tea
*Clausena anisata* (*Wild) Benth.*	Rutaceae	MW-059	Limch	Tree	Stem	Bone dislocation	Stem powder boiled with butter applied daily
					Whole plant	Mental illness/exorcism	The juice of whole plant is employed for bathing
*Clematis simensis Fresen.*	Ranunculaceae	MW-009	Azo Hareg	Climber	Leaf	Hemorrhoids	Drying the leaf then crushing it and mixing with butter finally apply to area once
					Leaf	Skin cancer	Crushing the leaf and apply it to the area of infection
					Leaf	Eczema	Drying the leaf, crushing it and then mixing it with benzene and wood charcoal
*Cordial africana Lam*.	Boraginaceae	MW-058	Wanza	Tree	Leaf	Nightmare	Powder of the semi-parasite worn as amulet against startling dreams
*Coriandrum sativum* L.	Umbelliferae	MW-052	Dinblal	Herb	Seed	Menorhagia	Handful of seeds from each pounded with onions and taken once mixed with milk of black cow
*Croton macrostachyus Del*.	Euphorbiaceae	MW-035	Bisana	Tree	Leaf	Malaria	Crushing leaf and drink with either *Guizotia abyssinica* (L.F.) *Cass*. or milk
					Root	Tuberculosis	Root powder taken pasted with honey or taken dissolved with mead
*Cussonia ostinii Chiov*.	Araliaceae	MW-048	Getem	Tree	Bark	Syphilis	An inside part of the bark is pounded into powder which is then taken once mixed with a glass of local beer
*Cynoglossum coeruleum Hochst. A. Rich. DC.*	Boraginaceae	MW-010	Shingug	Shrub	Leaf	Acute febrile illness	Crushing the leaf with fresh water
*Datura stramonium* L.	Solanaceae	MW-054	Astenagir	Shrub	–	Headache	Unspecified part of the plant pounded mixed with ink and placed under the skin of the head
					Leaf	Mumps	Fresh leaf is tied on to the site of the problem
*Dovyalis* *abyssinica* *(A. Rich.) Warb.*	Flacoutiaceae	MW-011	Koshim	Tree	Seed	Decayed teeth	Brushing the decayed teeth with the yellow seed of the plant
*Echinops kebericho Mesfin*	Asteraceae	MW-012	Kebericho	Shrub	Stem	Evil eye	Drying, crushing and adding the seed on fire to smell
					Stem	Tape worm	Drying and crushing then drink by mixing with *Capsicum annuum* L. and salt
					Stem	Common cold	Burning the root and inhale it
					Stem	Acute febrile illness	Burning the root on fire and fumigate
*Embelia schimperi Vatke*	Myrsinaceae	MW-013	Enkoko	Shrub	Seed	Anthrax	Crushing the seed with the seeds of *Guizotia abyssinica* (L.F.) *Cass*. and eat with honey
					Seed	Tape worm	Crushing the seed and drink with alcohol
*Englerina* *woodfordioides* (*Schweinf.) M.* *Gilbert*	Loranthaceae	MW-014	Teketsila	Shrub	Leaf	Cutaneous leshmaniasis	Crushing the leaf and apply it topically
*Eragrostis tef (Zucc.)Trotter*	Gramineae	MW-045	Nech teff	Herb	Seed	Diarrhea	Porridge of the floor eaten three times daily
*Erytbrina brucei Schweinf*.	Fabaceae	MW-015	Korch	Tree	Leaf	Wound	Crushing its leaf with the leaves of *Solanum incanum* L. and *Phytolacca* *dodecandra L’Hérit*, finally apply it to the wound once for 3 days
*Eucalyptus globulus Labill*.	Myrtaceae	MW-016	Nech Bahirzaf	Tree	Leaf	Common cold	Burning the leaf on fire and inhale it
*Euphorbia abyssinica* *J.F.Gmel.*	Euphorbiaceae	MW-034	Kulkual	Tree	Latex	Jaundice	Mixing the latex with teff powder and putting it in fire till it becomes semidry
					Root	Rabies	Crushing the root and mixing with powder of wheat or teff, finally drying it on fire
					Leaf	Cutaneous leshmaniasis	Crushing the leaf and mixing it with butter
					Latex	Skin cancer	Applying the latex to the affected area
					Root	Malaria	Crushing the root and drink with milk
*Ficus vasta Forssk*.	*Moraceae*	MW-038	Shola	Tree	Bark	Epilepsy	Fumigate the patient once daily with the smoke of the powder of bark
					Root	Frequent miscarriage	Root and leaf powder taken once mixed with milk
*Grewia ferruginea Hochst. ex A. Rich.*	*Tiliaceae*	MW-017	Lenquata	Shrub	Bark	Hair fungus	Washing hair with the latex of the bark
*Guizotia abyssinica* (L.F.) *Cass.*	*Compositae*	MW-040	Nug	Herb	Fruit	Rabies	A cup of oil is given in morning in empty stomach for 3 days
					Seed	Dry cough	A cup of seed powder decoction is given orally in the morning and evening for a week
					Seed	Retained placenta	Boil the oil of the seed together with onions and egg and allow the steam to get into the vagina cavity
*Hagenia* *abyssinica (Brace.)* *J.* *F.* *Gmel.*	*Rosaceae*	MW-018	Koso	Tree	Leaf	Tape worm	Crushing the fresh leaves and mix with water and drink it once
*Hordeum vulgare*	*Gramineae* a	MW-033	Gebs	Grass	Seed	Peptic ulcer disease	Drying the seed on fire and eat
*Hypericum revolutum* (*Forssk.) Vahl*	*Guttiferae*	MW-056	Amja	Shrub	Fruit	Earache	Fruit juice applied as ear drops
*Juniperus procera Hochst*.	Cupressaceae	MW-057	Tsid	Tree	Resin	Congestive heart failure	Powder of fried resin taken orally mixed with water
*Justicia* *schimperiana* (*Hochst.ex* *Nees) T. Anders.*	Acanthaceae	MW-019	Simeza	Tree	Leaf	Anthrax	Crushing the leaf and mix with fresh water drink it once on empty stomach
*Kalanchoe* *petitiana A.Rich.*	Crassulaceae	MW-020	Andahula	Herb	Stem	Hemorrhoids	Putting the stem on fire till it becomes hot then putting it on the area of infection
					Leaf	Abdominal cramp	Crushing the leaf and eat it
					Root	Sexual dysfunction	Milk decoction of the fresh pulverized roots and leaves
*Lathyrus sativus* L.	Leguminosae	MW-044	Guaya	Herb	Seed	Constipation	Seeds eaten cooked at least once daily to overcome evacuation problem
*Lepidium sativum* L.	Cruciferae	MW-050	Feto	Herb	Seed	Wound	Powder of seed mixed with latex of *Euphorbia abyssinica* and bandaged once daily every other day
*Linum usitatissimum* L.	Linaceae	MW-021	Telba	Shrub	Seed	Anthrax	Crushing the seed with the seeds of *Lepidium sativum* L. or *Guizotia abyssinica* (L.F.) *Cass.* Then eating with honey on empty stomach
					Seed	Peptic ulcer disease	Boil the seed with water and after cooling drink it
					Seed	Pain during delivery	Putting the seed in water till it becomes semisolid and drink it
*Lupinus albus* L.	Fabaceae	MW-041	Gibto	Herb	Seed	Hypertension	Small quantity of seed and fruit is grounded with water, filtered. The resultant juice is given orally in the morning for 1 month
*Myrica salicifolia Hochst. ex A.Rich*	Myricaceae	MW-060	Shinet	Tree	Root	Headache	Butter paste of the root powder placed in the nostril
*Olinia rochetiana A. Juss.*	Oliniaceae	MW-023	Chife	Tree	Leaf	Teeth ache	Chewing the leaf within the mouth for about a minute and spit it
					Root	Goiter	Powder of root and leaf mixed with latex of *euphorbia abyssinica* and bandaged on the goiter once every other day
*Osyris quadripartite Decn.*	Santalaceae	MW-042	Keret	Tree	Leaf	Jaundice	A handful of fresh leaf is grinded and cup of this juice given orally for 15 days
*Phytolacca* *dodecandra L’Hérit*	Phytolaccacee	MW-024	Mekan Endod	Climber	Leaf	Anthrax	Crushing the leaf with fresh water and drink one glass of it once
					Leaf	Scabies	Crushing the leaf and washing the area of infection with the crushed leaf
					Bark	Jaundice	Powder of bark taken once mixed with diluted local beer
					Root	Rabies	Crushing the root and drink with honey
*Rhamnus prinoides L’Herit*	Rhamnaceae	MW-026	Gesho	Shrub	Leaf	Wound	Crushing the leaf and apply it to the wound till the wound cures
					Leaf	Epitaxis	Leaf powder taken mixed with once own urine
*Ricinus communis* L.	Euphorbiaceae	MW-047	Chakma	Shrub	Leaf	Appendicitis	Fresh pulverized leaf infused in water solution of safflower powder and one glass taken only once
*Rumex nepalensis Spreng.*	Polygonaceae	MW-027	Yewusha Milas	Herb	Leaf	Acute febrile illness	Crushing leaf with fresh water and wash with it
*Rumex nervosus Vahl*	Polygonaceae	MW-028	Ambacho	Shrub	Leaf	Wound	Crushing the leaf and mixing with benzene, then boil it, finally washing the wound with it
*Ruta chalepensis* L.	Rutaceae	MW-029	Tiladam	Shrub	Seed	Evil eye	Crushing the seed with the seeds of garlic and apply it on the nostril
					Leaf	Epitaxis	Fresh pulverized leaves are placed in the nostril
					Root	Headache	Powder of root and garlic mixed with water
					Root	Recurrent seasonal illness	Decoction of fresh pulverized root applied externally
*Skebergia capensis Sparrm.*	Meliaceae	MW-046	Lol	Tree	Bark	Malaria	Infusion of fresh pulverized bark
*Snowdenia polystachya (Fresen.)Pilg.*	Graminaceae	MW-051	Muja	Herb	Whole plant	Menorhagia	Juice or infusion of whole plant taken once
*Solanum incanum* L.	Solanaceae	MW-055	Embuay	Climber	Seed	Attention deficient disorder	Powder of seed given in small amount through the nose to help a child to be a fast learner and intelligent
*Syzygium guineense (Wild.) Dc.*	Myrtaceae	MW-043	Dokma	Tree	Root and leaf	Syphilis	A decoction is made from each one teaspoon of root and leaf powder and a cup of this decoction is given orally three times a day for 7 days
*Trigonella foenum*-*graecu*	Fabaceae	MW-037	Absh	Herb	Seed	Peptic ulcer disease	Putting in water, drying it, crushing and the eating by mixing with water and sugar
					Seed	Dry cough	Crushing the seed and boil with milk
					Seed	Weight loss	Putting in water, drying it, crushing and the eating by mixing with water and sugar
*Descopodium penninervum Hochst.*	Solanaceae	MW-022	Aluma	Tree	Seed	Wound	Crush the dried seed and apply the powder to the affected area for 3 days
*Urtica simensis*	Urticaceae	MW-025	Sama	Herb	Leaf	Peptic ulcer disease	Boil the semi-crushed leaf and eat it for 2 or 3 days
					Root	Malaria	The root will be crushed and dried the mixed with fresh water, drink one glass of it and drink much amount of milk
*Vernonia amygdalina Del.*	Asteraceae	MW-030	Girawa	Tree	Leaf	Bladder distention	Crushing the leaf with water and drink about one glass once
*Zehneria* *scabra* *(Linn.* *f.) Sond.*	Cucurbitaceae	MW-031	Haregresa	Climber	Leaf	Diarrhea	Crushing the leaf and mix with some fresh water, finally drink one cup of it
					Leaf	Acute febrile illness	Boil the leaf in water till it evaporates and then fumigate with it
					Leaf and root	Sexual dysfunction	Bathe in the infusion of leaf and root for 7 days
					Root, bark and leaf	Gout	Decoction of root, bark and leaf and excrement of hyena employed for bathing
*Zingiber officinale Roscoe.*	Zingiberaceae	MW-032	Zingibl	Bulb	Root	Bladder distention	Crushing the root with fresh water and drink about one glass once
					Root	Abdominal cramp	Crushing the root and mixing with some water then drink the filtrate

### Plant parts used

**Fig. 3:**
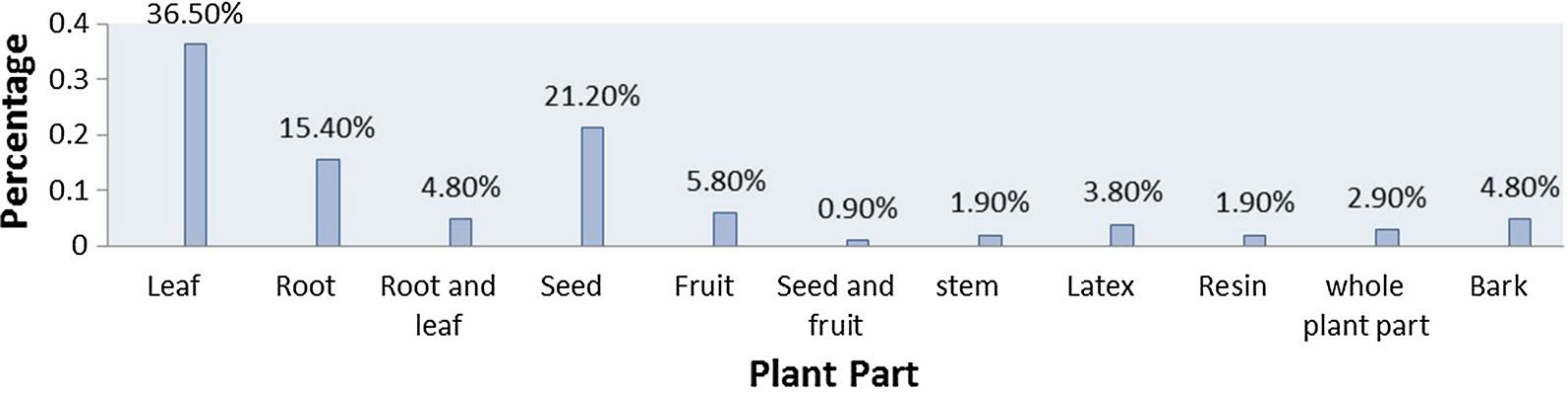
Frequency distribution of plant parts used to prepare remedies

According to this survey, the commonly used plant part was leaf (36.5%), followed by seed (21.2%) (Fig. 3).

### Method of preparation, routes of administration and dose

Traditional medical practitioners used simpler techniques like crushing and powdering with the help of easily available materials like water, honey and milk for preparation of remedies to treat various human ailments as shown in Table 1. This survey also documented that most of the remedies were given orally, (44%). Topical (26.5%), nasal (25.5%), rectal (2%), vaginal (1%) and subcutaneous (1%) routes are also used. This study showed that TMPs in the district were not aware of the exact dose of remedies to be administered. They easily determined the dose depending on mainly age. The doses of 24 preparations were not determined. Healers expressed doses as a glass of, half a cup of or a teaspoon full of.

### Dosage forms and frequency of administration

**Fig. 4:**
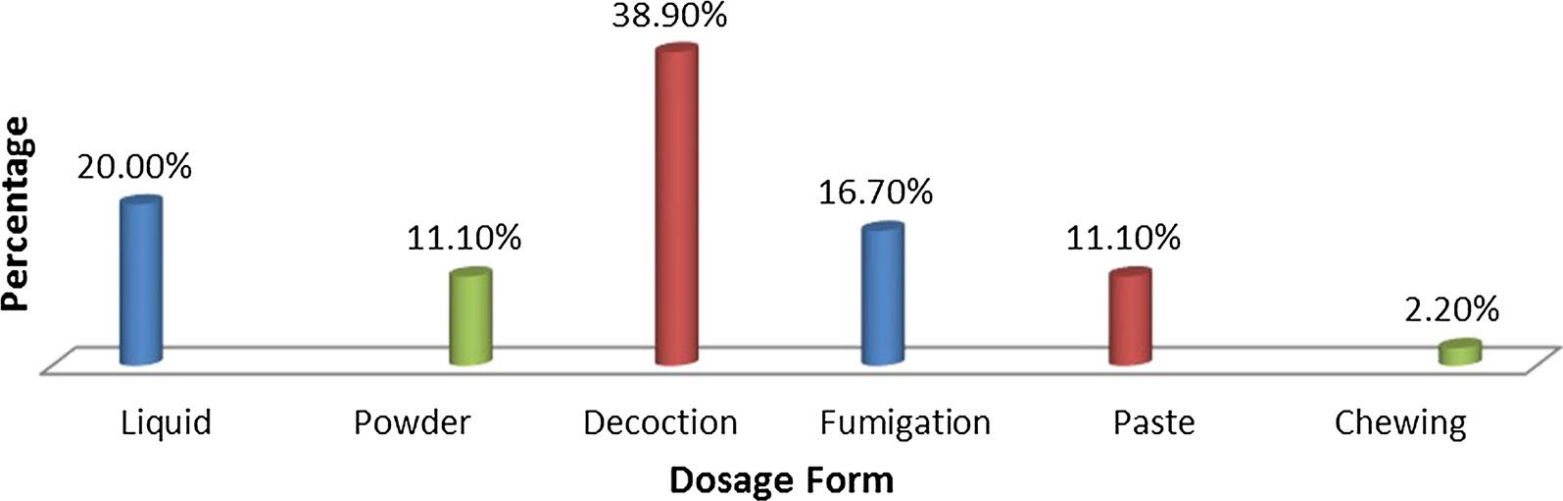
Frequency distribution of dosage forms of plant remedies

**Fig. 5:**
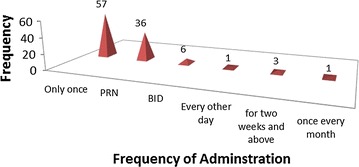
Distribution of frequency of administration of plant remedies

The documented 60 species of medicinal plants were reported to be formulated in various forms. Majority of dosage forms were decoctions 35 (38.9%) followed by liquid preparations 18 (20%) as shown in Fig. 4. Most of the preparations were given only once (Fig. 5).

### Solvents and additives

Forty-three percent of the formulations did not require any additive or solvent. Of the formulations that involve the use of solvents, water accounted 25 (42.4%) followed by milk 8 (13.6). Different additives like butter, honey, sugar and others were also incorporated (Table 2).

**Table 2:** Solvents and additives used

Solvents and additives	Number (%) of formulations
Water	25 (42.4)
Alcohol	5 (8.5)
Milk	8 (13.6)
Benzene	2 (3.4)
Honey	4 (6.8)
Salt	1 (1.7)
Sugar	4 (6.8)
Charcoal	1 (1.7)
Butter	5 (8.5)
Ink	1 (1.7)
Urine	1 (1.7)
Teff	2 (3.4)

### Contraindications and side effects

According to TMPs of the area, 42 (40.4%) of the formulations were contraindicated for pregnant patients. No contraindication was indicated for 35 (33.6% of the formulations (Table 3). Twelve (11.5%) of the preparations were free from any side effect (Table 4).

**Table 3:** Distribution of contraindication to remedies

Category	Frequency	% Age
Contraindicated to		
Children	19	18.3
Pregnant women	42	40.4
Elderly	8	7.7
No contraindication	35	33.6

**Table 4:** Frequency distribution of side effects of remedies

Category	Frequency	% Age
Those with known side effects		
Nausea	15	14.4
Vomiting	15	14.4
Diarrhea	8	7.7
Loss of consciousness	8	7.7
Local pain	12	11.5
Headache	6	5.8
Nasal stiffness	10	9.6
PUD	4	3.8
Constipation	3	2.9
Abdominal cramp	9	8.7
Free from any side effect	12	11.5

### Drug food interactions

According to this study, only 17% of the formulations possessed drug food interactions, of which 12 (66.7%) were exhibited by preparations for gastrointestinal problems. Synergistic reactions were observed in poly herbal preparations like in the case of remedies for evil eye.

### Storage

**Fig. 6:**
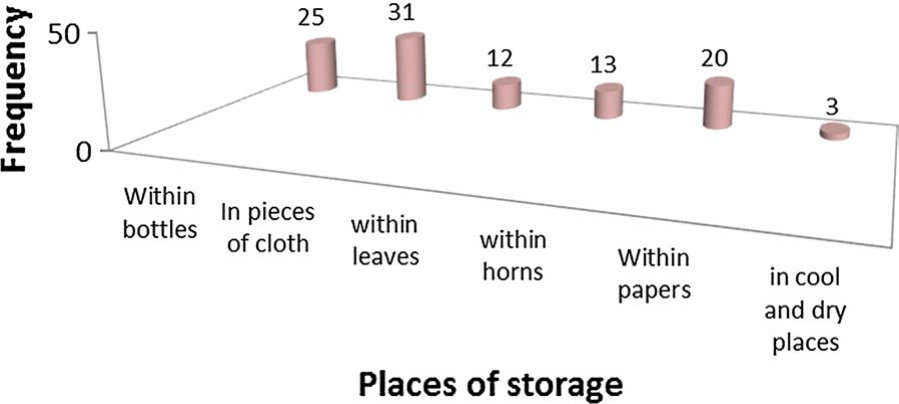
Frequency of storage of remedies

Asked on how they store plant remedies, healers responded that they don’t normally store plant preparations; rather they collect fresh material and formulate remedies. For those medicinal plants which are not easily available and which are seasonal, they collect and store in papers, horns or and with in bottles. Only three percent of the total preparations were stored in cool and dry places (Fig. 6).

### Informant’s consensus

Depending on the data obtained, seven use-categories (Table 5) were set in which 127 use reports were documented. As depicted below, informant consensus factor values and the mean ICF are close to 1. There is high uniformity in plant consumption for respiratory diseases.

**Table 5:** Informants’ consensus score

Use category	Species (#) (n_t_)	Use-reports (Ur)	ICF (n_ur_ − n_t_/n_ur_ − 1)
Gastrointestinal problems	7	15	0.57
Central nervous system disorders	9	18	0.53
Respiratory problems	2	6	0.8
Urinary tract problems	7	15	0.57
Skin problems	8	13	0.42
Cardiovascular disorders	13	28	0.55
Other organ problems	14	32	0.58
Mean ICF			*0.57*

### Fidelity level

In the survey, the FL values were analyzed for seven plants in treating three medical conditions (Table 6). *Croton macrostachyus* (FL = 0.78) is reported by high number of informants (36%) in treating malaria. *Allium sativum* (FL = 0.75) is more preferable than *Echinops kebericho* (FL = 0.64) in treating evil eye.

**Table 6:** FI for plant species used to treat evil eye, malaria and rabies

Ailments	Percentage of informants	Species	Np	N	FI (Np/N)
Evil eye	30	*Allium sativum*	6	8	0.75
	34	*Echinops kebericho*	7	11	0.64
Rabies	9	*Guizotia abyssinica*	4	5	0.8
	34	*Phytolacca* *dodecandra*	8	9	0.89
Malaria	19	*Urtica simensis*	10	15	0.67
	36	*Croton macrostachyus*	7	9	0.78
	5	*Euphorbia abyssinica*	5	12	0.42

*Np* number of TMPs who used the medicinal plant for the same purpose, *N* number of TMPs who used the medicinal plant for various purposes

### Threats to medicinal plants

As shown in Table 7 below, drought, overgrazing and firewood collection are the major threats of medicinal plants. Practitioners leveled drought as the most serious threat.

**Table 7:** Threats to medicinal plants

Threats to medicinal plants	% of TMPs
Drought	55.6
Overgrazing	22.2
Firewood collection	15.6
Agricultural expansion	4.4
Soil erosion	2.2

## Discussion

This study revealed that about 60 plant species find applications by the TMPs of the woreda. Those plants were identified and distributed in 42 families. Families, Graminae and Solanaceae each accounts 4 (9.5%) medicinal plants followed by Fabaceae and Leguminosae, 3 (7%). But Fabaceae was the dominant family according to the conducted in Hawasa [13], Wayu Tuka District of Oromiya region [14] and Benshangul-Gumuz [15]. In addition a study done in Spain [16], Korea [17] and Loma and Gena Bosa Districts [18] showed that Asteraceae has the highest number of medicinal plants. Caesalpiniaceae was the family with higher number of plants according to the study in Nigeria [19].

The ailments reported to be handled by the TMPs of the district are those disorders most prevalent in the district. According to the result of this study, the majority of plants were reported to treat wound followed by malaria, evil eye and anthrax. However, a study done in Hawasa [13] showed that stomach ache is the disease treated by large number of medicinal plants. In addition a study done in Sheko ethinic group of Ethiopia [8] showed that skin and gastrointestinal problems were the commonly treated diseases. According to a study in Lebanon [20], most medicinal plants were used to treat gastrointestinal disorders, kidney and urinary diseases as well as blood and cardiovascular diseases. Diarrhea was the commonly treated disease in Benshangul-Gumuz [15].This discrepancy may be the result of the difference in the climatic, ethnic, and hygienic conditions the areas. The current study is also unlike the one done in Israel [21]. Mental illnesses were commonly treated according to the study in Kenya [22].

The most commonly used plant part was leaf in this study area which is in agreement with other studies. [8, 13, 15, 18, 19, 21, 23–26]. Considerable threat to the mother plant radiates to the various parts of the plant. However, root was the commonly used plant part according to a study done in Benshangul-Gumuz [15]. Medicinal plants were formulated in various forms using various solvents and additives. They were formulated as decoctions, liquid preparations and pastes. This is supported by a study done in Korea [17], Israel [21], Gondar zuria woreda [24] and Hawasa [13]. However, in a study done in Chencha [18] and Tewledere districts [27], the majority of remedies were formulated as concoctions. Practitioners prepare remedies in such a simple manner without further processing which may be due to lack of education and processing instruments. TMPs of the current study area used butter, charcoal, sugar, milk and salt as additives to increase the efficacy and potency of the remedies. The rationale behind the use of honey and sugar is just to make the formulation palatable. This is supported by a study done in Israel [17] and Hawasa [13].

This study showed that there was no harmony in measurement or unit used among practitioners. Most informants stated measuring units like cup, spoon, bottle and handful which lack accuracy. This problem was also observed in studies done elsewhere which may be due to lack of education [13, 14, 19, 28].

This study revealed that higher sizes of preparations were given orally which agrees with results of other studies [11–13, 15, 17, 22–25, 27–29]. Practitioners prefer simple routes like topical and oral due to their inability to administer remedies in other routes like intravenous. Oral administration allows relatively fast physiological action of remedies on pathogens and enhance its efficacy. However, studies conducted in Sheko ethnic group in Southwest Ethiopia [8] revealed that most preparations were prescribed for administration to the cutaneous route. According to the result of the current study, most formulations were given only once. This may be due to the fact that most practitioners do not know the actual dose to be given and fear the risk that comes at the end of the treatment due to over dose and continuous administration.

One-third of the medicinal plants recorded were trees. However, other research works indicate the abundant use of herbs [8, 22, 24, 25, 27, 30]. The availability of most woody plants in the area might have enforced the local inhabitants to rely on tress.

Most of the formulations were contraindicated for pregnant patients. This is due to the healers’ belief that it may harm the fetus. No contraindication is indicated for one-third of the formulations. Most preparations taken orally cause nausea, vomiting and abdominal cramp whereas, some of the preparations are free from any side effect which may be due to the lack of follow up of patients by healers once they gave remedies and due to illiteracy of the patients.

This study revealed that, only some of the formulations possessed drug food and drug–drug interactions, this may be because most practitioners are illiterate, they do not know about the interaction of their remedies with modern medicines. In addition practitioners do not follow the progress of their patients, hence have little information on drug food interaction. Synergistic reactions were observed in poly herbal preparations like in the case of remedies for evil eye.

According to this study, practitioners do not normally store remedies which is in contrast to the study done in Addis Ababa [28]. For those medicinal plants which are not easily available and which are seasonal, practitioners collect and store in papers, horns and with in bottles. Only three percent of the total preparations are stored in cool and dry places. This may be due to the lack of modern education about drug storage and other health related issues.

A total of 127 URs from 60 species of plants were recorded which were assigned to seven use categories. Analysis of ICF shows that there exists a high evenness of plant consumption among the population which is in harmony with the study in Chencha [18]. The low ICF for skin problems may because of a multifaceted preparation of plants required to treat disease. Majority of plant species have a number of medicinal values, which result in higher variety of use reports.

*Croton macrostachyus* (FL = 0.78) is reported by high number of informants (36%), hence more preferable than *Euphorbia abyssinica* (*0.42*) and *Urtica simensis* (0.67) in treating malaria. However, despite *Allium sativum* (FL = 0.75) is reported by less figures of informants (30%), than *Echinops kebericho* (FL = 0.64) which is mentionedd by relatively higher percentage of practitioners (34%), it seems that *Allium sativum* is more preferable than *Echinops kebericho* in treating evil eye. But a study done in Sheko [8] indicated that *Ocimum lamiifolium*, *Phytolacca dodecandra*, *Amaranthus dubius* and *Amaranthus graecizans* were the medicinal plants assigned with the highest FL values. This discrepancy may be the result of the differences in the type of diseases dominating the areas, and the level of availability of the medicinal plants.

According to the results of this study, drought is the most serious threat to medicinal plants followed by overgrazing. This is in conformity with the survey conducted in Gemad district [25] and Kilte Awulalo [27], but according to a study done in Loma and Gena Bosa [26], agricultural expansion was the major threat followed by timber and other demands. This is probably due to the increasing number of population. However, study done in Hawasa city [13] indicated urbanization as the most serious threat for medicinal plants.

## Conclusion

Dega Damot district is loaded in its medicinal plant diversity and indigenous knowledge though plants are highly affected by drought, overgrazing and firewood collection. Therefore awareness activities must be created among the district’s population by concerned governmental and nongovernmental organizations about the value of medicinal plants and conservation of these plants. The healing potential and associated adverse issues of the claimed medicinal plants should be assessed before proposing for a broader utilization.

## Additional file

**Additional file 1.** Semi-structured interview questions.

## Abbreviations

HIV: human immunodeficiency virus
ICF: informant’s consensus factor
PUD: peptic ulcer disease
TM: traditional medicine
TMPs: traditional medical practitioners
UR: use-report
