# Supplementary material for: Ethnopharmacologic survey of medicinal plants used to treat human diseases by traditional medical practitioners in Dega Damot district, Amhara, Northwestern Ethiopia
Source: BMC Res Notes. 2017 Apr 18;10:157. doi: 10.1186/s13104-017-2482-3 (PMC5395840; doi:10.1186/s13104-017-2482-3)
Supplement: Supplementary file 1 — Additional file 1. Semi-structured interview questions. [file 13104_2017_2482_MOESM1_ESM.docx]

**Semi-structured interview questions**

**TMP code**:_________

**Kebele**: ________________

1. **Socio-demographic data**
2. Age: -------------- (in years)
3. Sex: Male-------- Female---------
4. Marital status:
5. Married
6. Single
7. Widow
8. Divorced
9. Education:
10. Non
11. Primary
12. Secondary
13. University/College
14. Religious education
15. Occupation: ------------------------------------------------------
16. Religion:
17. Ethiopian Orthodox tewahdo
18. Protestant
19. Muslim
20. Others
21. What is the source of your knowledge?
22. Family
23. Religious Institutions
24. Preceding sickness and corresponding use
25. Apprenticeship
26. Gift of god
27. For how many years did you practice your traditional healing? ..................... (in years)

**II**. **Medicinal plants used by traditional medical practitioners and diseases treated**

| S.No | Disease treated | Local name of the plant | Part used | Dosage form | Method of preparation | Dose given | Route of administration | Frequency of administration | Side effect | Contraindication | Drug-drug**/** food interaction |
| --- | --- | --- | --- | --- | --- | --- | --- | --- | --- | --- | --- |
|  |  |  |  |  |  |  |  |  |  |  |  |
|  |  |  |  |  |  |  |  |  |  |  |  |
|  |  |  |  |  |  |  |  |  |  |  |  |
|  |  |  |  |  |  |  |  |  |  |  |  |
|  |  |  |  |  |  |  |  |  |  |  |  |
|  |  |  |  |  |  |  |  |  |  |  |  |

1. **Storage and threats of medicinal plants**
2. Where do you store medications?
3. Within bottles
4. In pieces of cloth
5. Within leaves
6. Within horns
7. Within papers
8. In cool and dry places
9. Others (specify)--------
10. What do you think the threats are for medicinal plants?
